# A comparative study on flavour components and therapeutic properties of unfermented and fermented defatted soybean meal extract

**DOI:** 10.1038/s41598-020-62907-x

**Published:** 2020-04-07

**Authors:** Kadry Z. Ghanem, Mohamed Z. Mahran, Manal M. Ramadan, Hassan Z. Ghanem, Mohamed Fadel, Mohamed H. Mahmoud

**Affiliations:** 1 Nutrition and Food Science Department, National Research Centre, Dokki Giza, Egypt; 2 Nutrition and Food Sciences Department, Faculty of Home Economics, Menufyia University, Menufyia, Egypt; 3 Chemistry of Flavour and Aroma Department, National Research Centre, Dokki Giza, Egypt; 4 Therapeutical Chemistry Department, National Research Centre, Dokki Giza, Egypt; 5 Microbial Chemistry Department, National Research Centre, Dokki Giza, Egypt; 6 Clinical Nutrition Department, Faculty of Applied Medical Sciences, Jazan University, Jazan, Kingdom of Saudi Arabia; 7 Deanship of Scientific Research, King Saud University, Riyadh, Saudi Arabia

**Keywords:** Gas chromatography, Cancer prevention

## Abstract

Microbial fermentation of plant material alters the composition of volatile and non-volatile plant natural products. We investigated the antioxidant, anticancer, and antiviral properties of extracts of defatted soybean meal fermented with *Aspergillus fumigatus F-993* or *A. awamori FB-133* using *in vitro* methods. Gas chromatography–mass spectrometry analysis of soybean meal fermented with *A. awamori FB-133* and *A. fumigatus F-993* identified 26 compounds with 11,14-octadecadienoic acid and methyl ester (63.63%) and 31 compounds with butylated hydroxytoluene (66.83%) and δ-myrcene (11.43%) as main constituents, respectively. The antioxidant activities of DSM extract were 3.362 ± 0.05 and 2.11 ± 0.02 mmol TE/mL, FDSM treated with *A. awamori FB-133* were 4.763 ± 0.05 and 3.795 ± 0.03 mmol TE/mL and FDSM treated with *A. fumigatus F-993* were 4.331 ± 0.04 and 3.971 ± 0.02 mmol TE/mL as determined by ABTS and FRAP assays, respectively. Both fermented extracts had better antioxidant activity than the unfermented extract as shown by multiple antioxidant activity assays. The concentration of fermented extracts required for 50% inhibition of cell viability was significantly lower than that of the unfermented extract when tested against the human liver cancer cell line HepG2 as shown by cell viability assays, indicating strong anticancer activity. The IC_50_ values for DSM, FDSM with *A. fumigatusF-993* and FDSM with *A. awamori FB-133* were27, 16.88 and 8.60 μg/mL, respectively. The extract of FDSM with *A. awamori FB-133* showed the strongest anticancer activity, compared to DSM and FDSM with *A. FumigatusF-993* extracts. Fermented extracts also reduced hepatitis A virus titres to a greater extent than unfermented extracts, thus showing strong antiviral property. Hepatitis A virus titres were reduced by 2.66 and 3 log_10_/0.1 mL by FDSM with *A. fumigatusF-993* and FDSM by *A.awamori FB-133*, respectively, compared to DSM (5.50 log_10_/0.1 mL). Thus, the fermentation of soybean meal with *A. fumigatusF-993* or *A. awamori FB-133* improves the therapeutic effect of soybean extracts, which can be used in traditional medicine.

## Introduction

Fermentation of plant materials causes a significant change in bioactive volatile and non-volatile components^1^. Moreover, the concentrations of both flavonoids and the unsaturated fatty acid esters, as well, increase due to the fermentation of soybean. Extracts of fermented soybean have shown strong antioxidant, anti-breast cancer and antiviral activities^2^. A growing major objective interest is to develop special catalytic systems (one-pot tandem), that integrate both the reactivity of a chemical catalyst and a selected active site of an enzyme^3^. Fungi (as yeasts, moulds), bacteria and several microorganisms are known to produce lipases that digest lipids. Moreover, fungal lipases are used as industrial catalysts to resolve racemic alcohols used for preparing some prostaglandins, steroids, and carboxylic nucleoside analogues. Such lipases also catalyse the hydrolysis of short chain triglycerides, which represent very good substrates for lipases. In addition, proteases and amylases, which catalyse the hydrolysis of proteins and carbohydrates respectively, are widely used in different industries. Recently, the biocatalytic potential of microbial lipases, in both aqueous and non-aqueous media, have encourage many industries to utilize these enzymes in a variety of great important reactions, especially organic synthesis reactions. The region selective nature of lipases are used for different purposes such as, fat modification, butter constituents, biofuels, synthesis of personal care products, the resolution of chiral drugs, flavour enhancers and synthesize of cocoa. Therefore, pharmacists, biophysicists, organic chemists, biochemists, biotechnologists, microbiologists and process engineers are now widely using lipases^4^. Although, soybeans are commonly rich in phenolics (e.g. flavanols, flavanones, phenolic acids, other phenolic compounds), yet isoflavones are the most abundant. However, such compounds can be modified through fermentation for improved antioxidant activities^5^. Fermentation of soybean with filamentous fungi has been reported to enhance their phenolic content and radical scavenging activity^6^. However, the antiviral activity of fermented soybean, for the treatment of viral diseases infecting human intestines, is not yet examined. Soybean fermentation can be done by using a number of microorganisms, for example fungi (as filamentous types) bacteria (as *Bacillus* sp. and lactic acid bacteria). Many fungal species, such as A. awamori, are commonly used for the production of many in fermented foods and flavours^7,8^ fermentation of soybean with *Aspergillus oryzae* increased its antioxidant activity^9^. Some *Lactobacillus* spp. and *Bifidobacterium* spp. have been reported to β-glucosides and enhance the isoflavone bioavailability by fermentation^10,11^. Several researchers have recommended the use of solid state fermentation method “SSF” for the production many bacterial and fungal metabolites, using different substrates^1,6,12^.

Cancer refers to the development of abnormal cells characterized by autonomous growth and loss of differentiation^13^. The American Cancer Society estimates the probable numbers of new cancer cases and deaths in the United States of America annually and compiles data on cancer incidence, mortality, and survival^14^. However, chemotherapy is often accompanied by adverse effects, which prolongs patient recovery^13^. Recently, there has been a more growing interest in the large-scale industrial utilization of biocatalysts in the production of fine chemicals and medicinal compounds^15^. In recent years, plant products such as flavonoids, terpenoids, and alkaloids have been studied for their varied pharmacological properties including antioxidant activity against free radicals that can cause cancer, inflammation, and diabetes^16^. The high concentrations of both polyphenols and volatile constituents in plant extracts account for their higher antioxidant activity^17–19^. Recently, plant extracts have been screened for anticancer activity to identify novel therapeutic strategies against cancer^20–22^.

The major causative pathogens of foodborne illness are viruses, followed by bacteria and parasites^23^. Several pure and crude plant derivatives and extracts are now being used as novel antimicrobial and/or antiviral against herpes simplex virus type 1 (HSV-1) infections^24^. In the present times, the anti-HSV-1 potential of plant extracts including phenolic compounds, polyphenols, peptides, isoprenoidal glycosides, pyranocoumarins and benzophenones have been evaluated^25^. Several fungal species of the genus *Aspergillus*, e.g. *A. niger* and *A. phoenicus* were found to increase b-glucosidase enzyme (BGL) yield, which plays a key role in biomass hydrolysis by producing monomeric sugars from cellulose-based oligosaccharides^12^. In view of the above facts, the present study evaluates the efficiency of *A. fumigatus F-993* and *A. awamori FB-133* in bioactive compound production from soybean meal and the antioxidant, anticancer, and antiviral potentials of the ethanolic extracts of defatted soybean meal (DSM) fermented by *A. fumigatus F-993* or *A. awamori FB-133*.

## Methods

### Raw material and chemicals

#### Defatted soybean

Egyptian defatted soybean was obtained from the Department of Medicinal and Aromatic Plants, Ministry of Agriculture, Cairo, Egypt.

### Microorganisms and culture conditions

*Non mycotoxin producing two fungi namely A. fumigates F-993* and *A. awamori FB-133* were obtained from Microbial Chemistry Laboratory (National Research Centre, Dokki, Cairo, Egypt) and cultured on potato dextrose agar slants at 30 °C for 72 h. The spore suspensions were prepared by adding 10 mL sterile water to slant cultures and gently scraping the culture with a sterile wire loop.

### Fermentation of defatted soybean

The fermentation was carried out under solid state fermentation in 250 mL Erlenmeyer flasks containing 5 g of defatted soybean moistened to 50% with distilled water and inoculated with 1 mL spore suspension (10^6^ spores). The cultures were incubated at 30 °C for 3 days for solid state fermentation^24^.

### Defatted soybean extraction

Volatile compounds were extracted from fermented defatted soy bean DSM samples according to previously reported protocols with some modifications^25,26^. The DSM and fermented DSM (FDSM) samples were ground individually to a powder using a coffee bean blender. Five grams of the ground powder was transferred into a 25 mL test tube and extracted with 15 mL ethanol. The supernatant was separated from the residue by centrifuging at 2000 × *g* for 10 min in a centrifuge and transferred to a clean test tube. The residue was re-extracted with 15 mL ethanol; the separated ethanol layers were combined, and dried using a vacuum evaporator at less than 50 °C. The dried soybean extract was weighed and stored at −20 °C.

### Gas chromatography–mass spectrometry (GC–MS) analysis

About 2 μl of each extract was used for GC–MS analysis using a HP 5890 GC coupled to HP5970 MS. The MS was set to an ionization voltage of 70 eV and the mass range of m/z 39–400 amu was scanned. The GC oven temperature was maintained initially at 50 °C for 5 min and then programmed to rise to 250 °C at a rate of 4 °C/min. Helium was used as the carrier gas at a flow rate of 1.1 mL/min. The injector and detector temperatures were set to 220 and 250 °C, respectively. The isolated peaks were identified by matching mass spectra with data in the National Institute of Standard and Technology library. Quantitation was carried out by peak area integration.

### Determination of antioxidant activity

#### 2,2-Diphenyl-1-picrylhydrazyl (DPPH) assay

The DPPH assay was performed according to a previously reported protocol^27^. The antioxidant activity was determined by a calibration curve prepared with ascorbic acid and expressed as mg of ascorbic acid equivalent (AAE)/mL of sample^28^.

#### 2,2′-Azino-bis (3-ethylbenzothiazoline-6-sulfonic acid) (ABTS) assay

The ABTS assay was performed as described previously^29^ and expressed in mMof Trolox equivalents (TE)/mL of extract. When the measured ABTS value was over the linear range of the standard curve, values of additional dilutions were recorded^30^.

#### Ferric reducing antioxidant power (FRAP) assay

The FRAP assay was performed according to the Benzie and Strain method, and expressed in mM TE/mL extract. Values of additional dilutions were measured when the FRAP value was over the linear range of the standard curve^9^.

### Anticancer activity

#### Cell culture and treatments

Human liver cancer cell lineHepG2 was obtained from the American Type Culture Collection (Rockville, MD, USA). Cells were grown in RPMI-1640 medium supplemented with 10% foetal bovine serum, 1% MEM non-essential amino acid solution, and 1% penicillin streptomycin solution (10,000 units of penicillin and 10 mg of streptomycin in 0.9% (NaCl) in a humidified atmosphere having 5% CO_2_ at 35 °C. The passage number range was maintained between 20 and 25. The cells were cultured in 75 cm^2^ cell culture flasks. For experimental purposes, the cells were cultured in 96-well plates (0.2 mL of cell solution/well). The optimum cell concentration, as determined by the cell line growth profile, was 2 × 10^5^ cells/mL. The cells were allowed to attach for 24 h prior to treatment with the extracts. The stock solution of each extract was filtered with 0.22 µm Minisart Filters (Sigma-Aldrich) before applying to cells. To test toxicity, 11 dilutions of each extract were tested by addition to cell monolayers washed with phosphate buffer saline (PBS)^10^.

#### 3-(4,5-Dimethyl-2-thiazolyl)-2,5-diphenyl-2H-tetrazolium bromide (MTT) assay

The MTT assay was performed using the protocol described previously^13^. Briefly, the cells were incubated for 4 h with 0.8 mg/mL of MTT dissolved in serum-free medium. After washing with 1 mL PBS, 1 mL DMSO was added and gently shaken for 10 min for complete dissolution. Aliquots (200 μL) of the resulting solution were transferred into 96-well plates and absorbance was recorded at 560 nm using the SpectraMax® 190 Microplate Reader (Molecular Devices). The concentration required for 50% inhibition of cell viability (IC_50_) was calculated as described^3^.

### Antiviral assay

#### Cells and viruses

Vero and BHK-21 cells were grown in Eagle’s minimal essential medium (MEM, GIBCO) containing 5% inactivated calf serum and 50 mg/mL gentamicin. Maintenance medium at pH 7.5 consisted MEM supplemented with 1.5% inactivated calf serum and gentamicin. The vesicular stomatitis virus (VSV) was propagated in Vero cells. Virus stocks were plaque-assayed on Vero cells as described^31^.

### Cytotoxicity assay

Vero cells grown in 96-well tissue culture plates were incubated with 12 concentrations (μg/mL) of each extract for 24 h at 37 °C. Cell viability was measured with the MTT assay. The cytotoxicity of each extract was expressed as the 50% cytotoxic concentration (CC_50_), which is the concentration required to reduce cell viability to 50% of the control^32^.

### Statistical analysis

The results are reported as mean ± standard deviation (SD) for at least three experiments. Statistical differences were analysed by the one-way ANOVA test.

## Results

### GC-MS analysis of FDSM

GC-MS analysis of extract of DSM fermented with *A. fumigatus F-993*(FDSM1) identified31 compounds, of which butylated hydroxytoluene (66.83%) and δ-myrcene (11.43%) were the main constituents (Table 1). In the GC-MS analysis of extract of DSM fermented with *A. awamori FB-133* (FDSM2), 26 compounds were identified, of which 11,14-octadecadienoic acid and methyl ester (63.63%) was the main constituents (Table 2).

**Table 1:** GC–MS analysis of defatted soybean meal fermented with *Aspergillus fumigates F-993*.

Identified compounds	Rt	Area%
3-Tetradecanol	5.32	0.49
Methyl-deacetyl colchicine	6.02	0.57
N-(butyl-1,2,3,4-tetrahydro-2-naphthyl)-hexa-methylen-imine	6.25	0.65
[3-(2-Cyclohexylethyl)-6-cyclo-pentyl-hexyl]-benzene	6.45	0.76
3-Dotriacontane	8.42	0.52
Cantaxanthin	8.73	0.50
Butanoic acid, heptafluoro-,methylester	8.81	0.52
1,5,5-Trimethyl-6-[2-(2-methyl[1,3]-dioxolan-2-yl)-vinyl]-4-methylene-7-oxabicyclo-[4.1.0]-hept-2-ene	8.86	0.60
δ-Myrcene	10.34	11.43
1,3-Dioxaphosphorinane-4,4,6-tri-methyl-2-phenoxy-2-oxide	10.54	1.72
[4,5b]-imidazole,1-formyl-3-ethyl-6-ádribofuranosyl-pyrazole	12.42	0.85
1,1′-[3-(2-phenyl-ethylidene)-1,5-pentanediyl]-benzene	13.49	0.74
1,4-Benzenedicarboxylicacid,[4-(methoxy carbonyl)-phenyl]-methyl ester	13.55	0.65
Methyl-6-oxoheptanoate	18.59	0.52
2(3H)-Furanone, dihydro-3,5-dimethyl	21.61	0.51
1,3,5-Trichloro-2,4,6-tris (4-nitro phenyl ethynyl)-benzene	21.77	0.63
Butylated hydroxytoluene	26.32	66.83
t-Butyl-ester of S-5′-Hydroxy dehydrothiojasmonic acid	27.57	0.75
Ethyl iso-allocholate	34.51	0.51
7,8-Epoxylanostan-11-ol-3-acetoxy	39.08	0.50
N-Methyl yunaconitine-3-ol	44.61	0.58
α-D-Glucopyranoside methyl-2,3-bis-(tri-methylsilyl),cyclic methyl-boronate	45.94	0.91
2,2′,7,7′-Tetra bromo-9,9′-spiro bi fluorenone	45.99	0.72
9,12,15-Octa decatrienoic acid, 2-phenyl-1,3-dioxan-5yl ester	46.05	0.64
Flavone- 4′-OH, 5-OH, 7-dioglucoside	58.07	1.07
Octadeca methyl cyclonona siloxane	59.77	0.76
Acetylcodeine	61.17	0.50
3,9-Epoxy pregnane-11,14,18-triol-20-one,16-cyano-3-methoxy,11-acetate	61.52	1.05
3,5-Dibromo-4-amino biphenyl	61.62	0.61
Dimethoxy glycerol docosyl ether	62.40	0.78
1,4,10,13-Tetra oxa-7,16-diaza-cyclo octa-decane,7,16-bis-(1-oxodecyl)	63.07	0.75

**Table 2:** GC-MS analysis of defatted soybean meal fermented with *Aspergillus awamori FB-133*.

Identified compounds	Rt	Area%
2-Nonadecanone	5.46	0.78
3,4-Pyridinediamine-6,6′-(1,3-phenylene)-2,5-diphenyl	6.88	0.77
[(4-Phenyl-5-sulfanyl-4H-1,2,4-triazol-3yl)-methoxy]-acetic acid	9.80	1.07
α-Bisabolene	10.33	1.47
5-Bromo-3,3″,4,4″-tetra butyl-2,2′,5′,2″-terthiophene	10.49	1.57
4-Bromo phenyl-bis-(2,4-dibromo phenyl)-amine	11.06	0.70
Di-2-benzothiazole disulfane	11.18	0.70
11-Octadecenal	12.39	0.92
Methyl di-homo ç-linolenate	13.63	0.68
Androst-5-en-4-one	25.82	0.93
Iso-humulone	26.96	0.79
11,14-Octadecadienoic acid, methyl ester	29.11	63.63
AnodendrosideA	33.08	0.78
2,2′-dioxospirilloxanthin	33.74	0.78
D-prim-cortisone	35.02	0.91
Rhodopin	35.55	0.69
Brom thymol Blue	41.62	0.77
Imidazole-2,4,5-d3	53.40	0.77
3,5-Dihydroxy cholestan-6-one	55.39	0.87
Anodendroside G	55.51	0.96
Stearic acid,3-(octadecyloxy) propyl ester	58.21	1.09
Rhodovibrin	58.75	0.71
Dimethoxy glycerol docosyl ether	61.17	1.13
Lucenin 2	62.36	1.09
Lycoxanthin	62.92	0.72
Ponasteroside A	63.66	1.27

### Antioxidant activity

The mean values of the antioxidant activity measured for DSM, FDSM1, and FDSM2 are presented in Table 3. The antioxidant activity of DSM extract was 3.362 ± 0.05 and 2.11 ± 0.02 mmol TE/mL as determined by ABTS and FRAPS assays, respectively, and 0.511 ± 0.01 mg AAE/mL with the DPPH assay. The antioxidant activity of FDSM2 extract was 4.763 ± 0.05 and 3.795 ± 0.03 mmolTE/mL with ABTS and FRAPS assays, respectively, and 0.625 ± 0.02 mg AAE/mL by DPPH assay. The antioxidant activity of FDSM1 extract was 4.331 ± 0.04 and 3.971 ± 0.02 mmolTE/mL by ABTS and FRAP assays, respectively, and 0.692 ± 0.01 mg AAE/mL with DPPH assay. Therefore, both FDSM extracts showed stronger antioxidant activity than the DSM extract.

**Table 3:** Antioxidant activity of extracts of defatted soybean meal(DSM) and defatted soybean meal fermented with *Aspergillus fumigatus F-993* (FDSM1) and *A. awamori FB-113* (FDSM2) measured by ABTS, FRAP and DPPH assays.

Extracts Extracts	ABTS (*mM TE/mL)	FRAP (*mM TE/mL)	DPPH (**mg AAE/mL)
DSM	3.36 ± 0.05^a^	2.11 ± 0.02^a^	0.511 ± 0.01^a^
FDSM2	4.763 ± 0.05^b^	3.795 ± 0.03^b^	0.625 ± 0.02^b^
FDSM1	4.331 ± 0.04^b^	3.971 ± 0.02^b^	0.692 ± 0.01^b^

All data are presented as mean ± SD. Values in the same column with the different superscripts are significant at P < 0.05. *Results are expressed as inhibitory activity in mmol Trolox equivalent/mL (mM TE/mL) of extract. **Results are expressed as inhibitory activity in mg ascorbic acid equivalent per mL (mg AAE/mL) of extract.

### Anticancer activity

Colorimetric MTT assay was used to determine the extracts’ anticancer activity, utilizing human hepatic cancerous cell line “HepG2”. The cytotoxic effects of DSM, FDSM2, and FDSM1 extracts, expressed asIC_50_, are summarized in f 4. The IC_50_ values for FDSM2, FDSM1, and DSM extracts were 8.6, 16.88, and 27 μg/mL, respectively, indicating that the FDSM extracts were more cytotoxic than the DSM extract.

### Antiviral activity

Table 4 also represents the titres of hepatitis A virus expressed as log_10_/0.1 mL. Extracts of FDSM1 showed stronger antiviral activity (2.66 log_10_/0.1 ml) compared to that of the DSM and FDSM2 extracts (5.5 and 3 log_10_/0.1 ml, respectively). The results of the present study revealed no significant differences in hepatitis A virus titres for FDSM1 and DSM extracts.

**Table 4:** Cytotoxicity and antiviral activity of extracts of defatted soybean meal (DSM) and defatted soybean meal fermented with *Aspergillus fumigatus F-993* (FDSM1) and *A. awamori FB-113* (FDSM2) against HepG2 liver cancer cell line and hepatitis A virus.

Extracts	Hepatitis A virus titre (log_10_/0.1 mL)	IC_50_ (µg/mL) for HepG2
DSM	5.50 ± 0.515^a^	27 ± 2.94^a^
FDSM2	3.0 ± 0.216^b^	8.60 ± 1.40^b^
FDSM1	2.66 ± 0.110^c^	16.88 ± 1.82^c^

All data are presented as mean ± SD. Values in the same column with the different superscripts are significant at *P* < 0.05.

## Discussion

Plant extracts contain many important phytoestrogens compounds, the most relevant to human health include: phytoestrogens genistein and daidzein (from soybean), formononetin (from clover), biochanin-A (from chickpeas) and coumestans and lignans (from flaxseed). However, the majority of such compounds are naturally found, in plants, in the glycosylated form. Moreover, the bioavailability of such glycoconjugates varies from their analogous unsubstituted aglycones. In mammals, it has been documented that phytoestrogens induce oestrogenic and anti-oestrogenic effects via their weak binding to the nuclear α and β oestrogen receptors (ER). In general, the relative binding affinity of phytoestrogens to β oestrogen receptors (ERβ) is higher than that to α oestrogen receptors (ERα). It has been reported that such phytoestrogens are associated with a lower rate of steroid-hormone-dependent cancers, such as colon, prostate and breast cancer.

It has been revealed that the role played by of polyphenols against flue virus, as antiviral mechanism, can be attributed to the inhibition of its adsorption to Madin-Darby canine kidney cells^33^, as well as the interference in viral membrane fusion^28^. It is a well-known fact that mechanism of oxidative stress, commonly associated viral infections including HSV-1, plays a major role in viral replication inside the viral-infected cells. Moreover, viral replication can be inhibited through restoring the intracellular redox conditions by the help of special antioxidants (e.g., glutathione “GSH” or *n*-butanoyl derivative “GSH-C4”) which inhibit HSV-1 replication^34,35^. In addition, Ramadan *et al*. 2014 have concluded that the main ingredients of the volatile compounds in DSM extracts are 23.8% (2,3-dicyano-7,7-dimethyl-5,6-benzodiene) and 19.1% (9-ditertbutyl-1-oxaspiro-4,5-deca-6,9-diene-2,8-dione)^1^.

It has been concluded that the major volatile antioxidant compounds, in FDSM, were butylated hydroxytoluene and 11,14-octadecadienoic acid, methyl ester. Octadecadienoic acid has been reported to be present in *Michelia champaca* Linn extracts and impart antioxidant and anticancer activities to the plant extract^36^. Specific food additives prevent lipid oxidation and rancidity, for example Butylated hydroxyl toluene “BHT”, which is the most commonly used synthetic antioxidant^37^. Studies have revealed that “BHT” has anticancer activities and tumour promotion effects. Moreover, its effect on other carcinogens depends on a number of factors including, the carcinogen, target organ, exposure parameters, as well as the animal being tested. The toxicity of butylated hydroxytoluene is suggested to be the result of it being an electrophilic metabolite^38–43^. Numerous studies have revealed that Butylated hydroxyl toluene “BHT” acts as a potent antioxidant that significantly inhibits cytokine-induced inflammatory responses in both human and mouse cells^44^.

Although, it has been concluded that the potency of modified ester of epigallocatechin gallate against HSV-1 infections, *in vitro*, is greater than that of epigallocatechin gallate, yet further studies are still recommended to fully explain the exact mechanism of action taking place in humans. Though, the use of the appropriate natural products could improve the health status of several patients via offering much better life. Murakami *et al*. 2003 have reported that bis-butylated hydroxyanisole inhibits the activation of activator protein-1, and hence it could be used in the chemoprevention of oral diseases, e.g. leukoplakia and destructive chronic periodontitis^45^.

The transformation of isoflavone glycosides into aglycones has been suggested to be promoted by soybean fermentation, hence fermented soybean is used as an ingredient in the formulation of foods with functional properties for health benefits^44^. By comparing with the results of earlier studies, it was clearly seen that these major compounds have major neuroprotective, antioxidant, anti-inflammatory, anticancer, hepatoprotective, and antimicrobial effects^46–50^. In conclusion, our results indicate that FDSMs possess antioxidant, anticancer and antiviral properties.
